# Eighth Annual Announcement of Medical Department Iowa University

**Published:** 1858

**Authors:** 


### EIGHTH ANNUAL ANNOUNCEMENT

OF THE

### COLLEGE OF PHYSICIANS AND SURGEONS,

OF THE

### IOWA STATE UNIVERSITY.

### BOARD OF TRUSTEES.

Hon. MATURIN L. FISHER, Pres't of the Board, ex-of.
Hon. ANSON HART, Secretary, Iowa City.
His Excel. JAMES W. GRIMES, Trustee, ex-officio.

Hon. GEO. W. McLEARY, Iowa City,

" ANSON HART, " "

" E. C. LYON, " "

" JAMES H. GOWER, " "

" G. D. PALMER, " "

} Terms expire in 1857.

" EDW. CONNELLY, " "

" H. W. LATHROP, " "

" M. J. MORSMAN, " "

" J. W. RANKIN, Keokuk.

} Terms expire in 1859.

" P. L. LAKE, Bellevue,
" LAURIN DEWEY, Mount Pleasant,
" THOS. FARMER, M'Kissack's Grove
" E. C. BIDWELL, Quasqueton,
" AMOS WITTER, Davenport,
} Terms expire in 1861.

Hon. LINCOLN CLARK, (to fill vacancy,) Dubuque, term expires in 1859.

### CURATORS.

Hon. Geo. G. Wright, Chief Justice of the Supreme Court.
W. S. Woodward, Associate " " "
L. D. Stockton, " " " "

| | | |
|---|---|---|
| Hon. Hugh T. Reed. | E. H. Harrison, Esq. | Col. Wm. Thompson, |
| E R. Ford, M. D. | Hosmer Curtis, " | Col. C. Perry, |
| Wm. Leighton, Esq. | Rev. Wm. H. Williams, | D. W. Kilbourne, Esq. |
| John M. Hyatt, " | Edw'd Kilbourne, Esq. | Hon. Charles Mason, |
| James L. Estes, " | Samuel F. Miller, Esq. | Hon. Hawkins Taylor, |
| Gen. V. P. Van Antwerp | Hon. R. P. Lowe, | Charles Parsons, Esq. |
| Rev. Prof. Elihu Gunn, | Rev. W. H. Cowles, | D. W. Ford, Esq. |
| Hon. James M. Love. | Col. Wm. Patterson, | A. L. Deming, Esq. |
| C. F. Conn, Esq. | Guy Wells, Esq. | J. M. Billings, Esq. |

### MEDICAL DEPARTMENT.

### AMOS DEAN, L. L. D.,

PRESIDENT OF THE UNIVERSITY.

### FACULTY.

JNO. R. ALLEN, M. D., Emeritus Professor of Obstetrics.

D. L. M'GUGIN, M. D., Professor of Physiology and Pathology, and President of the Faculty.

FREEMAN KNOWLES, M. D., Professor of Theory and Practice of Medicine.

J. C. HUGHES, M. D., Professor of Surgery and Dean of the Faculty.

A. S. HUDSON, M. D., Professor of Obstetrics and the Diseases of Women and Children.

WELLS R. MARSH, M. D., Professor of Chemistry and Toxicology, and Treasurer of the Faculty.

DANIEL MEEKER, M. D., Professor of Anatomy.

MONTGOMERY, JOHNS, M. D., Professor of Materia Medica, Theraputics and Microscopy.

HENRY STRONG, A. M., Lecturer on Medical Jurisprudence.

JOHN J. PAGE, M. D., Demonstrator of Anatomy.

J. WARD DAVIDSON, M. D., Prosector of the Chair of Anatomy.

---

SINCE the last, the seventh, session of this institution the Trustees and Faculty have, by a change in the distribution in the different departments, instituted another chair in addition to the six hitherto in the school. In accordance with this arrangement it will be seen by reference to the statement above of the organization of the Faculty, that Prof. A. S. Hudson, M. D., one of the original incorporators of the school, and for some years one of the Faculty, has been appointed to the chair of Obstetrics and Diseases of Women and Children, and Montgomery Johns, M. D., during the last session, lecturer on Physiology and general Theraputics, has been appointed to the chair of Materia Medica and Microscopy. The deserved popularity of Prof. Hudson, as a teacher during his former connection with the school, and his known ability and acquirements in medical science, render this acquisition one of great value.

With a Faculty thus complete in numbers, and made up of those ardently devoted to the practice of their profession and to the furtherance of the interests of medical science, the Trustees feel warranted in saying that the institution will enter upon its next annual session with every facility for making the course of instruction all that can be desired or found in any of the schools in the country.

---

### Free Lectures.

The American idea of making the education of its citizens one of the first duties of governments it is well known has been carried out in all of the new states by donation to them by the general government of school and university lands. The proceeds of these lands are made a permanent endowment to common schools and a university, the interest upon which is devoted to the maintenance of teachers and faculties in the same.

In the original organization of the medical department of the Iowa State University this consideration was given its due weight with the avowed intention of carrying out to the full the idea so soon as the pecuniary condition of the instituion should seem to warrant the step. In view of this, some years since, a system of scholarships, to be placed in the hands of certain state officers and by them conferred upon students seeking instruction in the state institution, was duly arranged and carried out. These scholarships entitled the holder to instruction in the institution at a price considerably reduced from that of those who were not in receipt of them. In this manner the benefits of the institution have been extended to many who must otherwise have been forced to forego them.

But the time having arrived when it seemed possible to carry out fully the original intentions, the Trustees have concluded to announce the *entire abolition of fees* except those for matriculation and graduation. In doing this they feel that they are but consenting to a demand of the public sentiment both within and without the profession, and that demand one in whose justice they heartily concur.

In view, however, of the reduction of expense of education to the student, it is the determination of the Trustees and Faculty to demand in return full compliance with the established requisitions, and furthermore it will be the constant aim of those concerned in the mânagement of the institution to assist in elevating the standard of these requirements until they shall be able to secure the unqualified approbation of the profession of the country.

---

### University Buildings, Apparatus &c.

The college buildings heretofore occupied having been found insufficient for the accommodation of the classes, a new brick edifice is in process of erection and it is intended to be ready for the opening of the ensuing session if possible. This building, sixty-five by ninety feet and three stories high, will contain two spacious lecture rooms, a commodious laberatory, dissecting room, museum and hospital room sufficient for thirty to forty beds.

Arrangements have also been made for the purchase of new appliances for teaching in the various departments. The chemical laberatory will be replenished with new apparatus sufficient, with that already in possession, to make the facility for teaching this science all that can be desired.

In practical anatomy the advanvages are ample. The supply of material is abundant, and every student is required to dissect one session. The personal supervision of the professor of anatomy, and the attendance of the Demonstrator, will afford every opportunity for improvement.

There is, in the college buildings, a museum, already valuable and yearly becoming more so. It includes numerous valuable and accurate botanical plates, a great variety of casts and models, including many wax preparations, a splendid suit of Materia Medica, abundant dry and wet anatomical preparations and morbid specimens and many interesting illustrations of natural history, Geology, &c.

To increase the number of surgical operations and induce patients from a distance to present themselves, all necessary arrangements have been made for their accommodation by the professor of surgery.

A large number of minor and capital surgical operations are performed before the class each year.

*All Surgical operations before the class are performed gratuitously.*

### Hospital and Dispensary.

The University Hospital was constructed by the city, in immediate cennection with the old college buildings, and was placed under the medical control of the faculty. It will be so arranged that students may enjoy equally all its advantages. Clinical instruction will be regularly given by the appropriate professor at this hospital.

In connection with the new college building, the faculty are about to establish a city dispensary for the purpose of securing further clinical instruction to the students, and every pains will be taken to afford opportunity for each to personally examine the cases prescribed for.

### Course of Lectures—Session of 1857-'8.

The regular course of lectures will commence on Thusrday, the 29th of Oct., and continue until the 1st of March. Six lectures will be delivered daily on the various branches of medical science. The public Introductory will be delivered on Thursday, P. M., by Prof. Hudson. The annual graduation exercises will be held, and the degrees conferred at the close of the term. Every student will be required, within two weeks after the opening of the session, to take his tickets and pay the regular fees.

The following are the requisites for the diploma :

1. The candidate must be twenty-one years of age—and present testimonials of a good moral character.

2. He must have attended two full courses of medical lectures; one of which must have been delivered in the medical department of the Iowa State University, or evidence of three years reputable practice will be regarded as equivalent to one course.

3. The candidate must have studied medicine three years, (including the courses of lectures) under the direction of a respectable medical practitioner.

4. He must furnish an original medical Thesis, (in his own hand writing, accompanied with his graduation fee,) which must be handed to the Dean, at least four weeks before the close of the session, and which shall, upon examination, prove satisfactory.

5. He must, at the close of the term, pass an examination satisfactorily to the faculty.

FEES, &c., &c.—The fee for the Diploma is $30 00 ; Matriculation ticket 10 00 ; the fee for admission into the dissecting rooms and demonstrations is $5 00. This covers the entire cost to the student.

Members of the profession from every part of the country, who are graduates of medicine, will, on presenting their diploma to the Dean, be admitted gratuitously to all the lectures.

Board can be obtained in the city at from $3 00 to $3 50. Students, upon arriving in the city, can obtain information as to good boarding houses, by calling at the office of the Dean, on Second street, near the St. Charles Hotel.

Medical books may be purchased at the book stores in our city, on as good terms as in any western city.

All letters of inquiry may be addressed to

J. C. HUGHES, DEAN of the Faculty.

### Graduates—Session 1856-'7.

At the close of the last session, Feb. 25, 1857, the degree of Dr. of Medicine was conferred by the President of the faculty on the following named gentlemen.

| NAME. | RESIDENCE. | SUBJECT OF THESIS. |
|---|---|---|
| ADAMS, GEO. G. | Pennsylvania, | Variola. |
| BALL, D. K. | Iowa, | Enteric Fever. |
| COONS, JAMES N. | Missouri, | Typhoid Fever. |
| COLINGS, ISAAC S. | Indiana, | Epidemic Dysentery. |
| ELSWORTH, H. N. | Indiana, | Veratrum Viride in Pneumonia and Typhoid Fever. |
| FLETCHER, R. J. | Kentucky, | Pulmonary Affections. |
| FRAZER, J. W. | Missouri, | Pneumonitis. |
| HARE, A. J. | Illinois, | Typhoid Fever. |
| JOHNSON, O. B. | Kentucky, | Dysentery. |
| KELLOGG, A. H. | Minnesota, Ter'y, | Premature Labor. |
| LEESE, JACOB | Missouri, | Pneumonia. |
| MEEKER, LYSANDER | Indiana, | Veratrum Viride, its Theraputic effets in Disease. |
| NELSON, G. W. | Iowa, | Nature in Miasm. |
| NEWELL, O. W. | Illinois, | Psycology vs. Pathology. |
| PYLE, ELIOTT | Iowa, | Diagnosis. |
| RUSSELL, W. A. J. | Illinois, | Pneumonia. |
| SLADE, J. W. | Illinois, | Duties of an Accoucher. |
| TAYLOR, COLUMBUS | Iowa, | Modes of Death. |

### Honorary Degree.

| | |
|---|---|
| Dr. P. Walker, | Iowa |
| Dr. George Shedd, | Iowa |
| Dr. M. W. Vroman, | California |
| Dr. S. A. Winslow, | Illinois |

### Catalogue of the Class—Session 1856-'7.

| NAMES. | RESIDENCE. | NAMES. | RESIDENCE. |
|---|---|---|---|
| Adams, Geo. G. | Iowa | Johnson, Oliver B. | Ky |
| Ayers, R. H. | Iowa | Jones, N. S. | Ohio |
| Barber, S. C. | Iowa | Kellogg, A. H. | M T |
| Barber, Samuel | Iowa | Kelley, J. T. | Ill's |
| Bartlett, W. H. | Iowa | Lees, J. J. | Mo |
| Ball, Daniel, R. | Iowa | Lade, A. J. | N Y |
| Bell, R. J. | Mo | Lewis Charles, | Iowa |
| Bailey Mahlon | Iowa | Little, Alex. C. | Ill's |
| Bryant, Jas. H. | Ind | Morgan Jonathan, | Iowa |
| Carter, J. A. | Iowa | Marion James | Ind |
| Cramer, C. C. | Ill's | Mason, F, | Ind |
| Coons, Jas. M. | Mo | Mason, John | Ill's |
| Collings, Isaac S. | Ind | Mallett, A. | Iowa |
| Copson H. | Iowa | Meeker, Lysander | Ind |
| Creele, C. M. | Ill's | McIntire, E. S. | Ill's |
| Davis, Eli C. | Mo | McGinnis, J. S. | Mo |
| Davis, M, | Iowa | McVey, John P. | Iowa |
| Davidson, I. W. | Iowa | McKean, C, A. | Iowa |
| Dewey, D. | Iowa | McMehan, W. S. | Iowa |
| Ellsworth, H. N. | Ind | Nelson, G. W. | Ill's |
| Edwin, E. | Iowa | Newell O. W. | Ill's |
| Ellyett, P. C. | Iowa | Osborne Henry | Iowa |
| Fenton, F. M. | Iowa | Porter W. M. | Mo |
| Frazer, J. W. | Mo | Pyle, Elliott | Iowa |
| Fletcher, J. Rob't. | Iowa | Purcell Wallace W. | Iowa |
| Gent, James C. | Ohio | Payne J. B. | Ill's |
| Githens, W. H. | Ill's | Russell, W. A. | Ill's |
| Givens, Wm. H. | Ill's | Russle, R. J. | Ill's |
| Henry, John E. | Mo | Ritchey, P., M. D. | Ill's |
| Holloway, Thos. A. J. | Md | Ritchey, Adlebert | Ill's |
| Hayes, R. W. | Ill's | Ray, J. L. | Ill's |
| Hall, Henry M. | Ill's | Stark, C. N. | Ill's |
| Jeffris, P. C. | Iowa | Skinner, A. O., M. D. | Iowa |
| Johns, J. K. | Md | Slade, John W. | Ill's |
| | | Slack, Josiah | Ill's |

| | | | |
|---|---|---|---|
| Slack, N. G. | Ill's | Woodworth, E. N. | Iowa |
| Stevens, A. T. | Mo | Whitted, N. W. | Iowa |
| Scoles, H. J. | Iowa | Wood, J. A. | Ind |
| Savill, Matt | Ind | Wright, L. W. | Ill's |
| Swearingen SamuelS. | Mo | Wilson, G. W. | Iowa |
| Showman, Thos. H. | Ill's | Wood, Adolphus G. | Mo |
| Taylor, N. | Mo | Williams, C, | Iowa |
| Taylor, Columbus | Iowa | Young, George B, | Iowa |
| Van Sicklin, Charles | Iowa | | |

### Notices.

To practitioners and student of medicine who may have received scholarships, we whould say that their presentation to the Dean at the opening of the session becomes unnecessary, as all students are received upon the paymant of the matriculation fee of $10 00.

The faculty of the medical department of the Iowa State University, would call the attention of the profession throughout the entire west, to the fact that all patients, medical or surgical, when coming before the class, will be operated upon or prescribed for gratuitously; and the profession would confer a favor upon the institution, and those connected with it, by sending cases which they may not wish to treat themselves. Our surgical cliniques have generally been large—as many as three cases of Lithotomy have been operated upon before the class during one session.

### Text Books.

Students would do well to furnish themselves with copies of some of the following works on the different departments.

Anatomy.—Morton; Wilson; Cruveilhier.
Chemistry.—Rand; Turner; Lehmann.
Institutes. — Kirkes; Carpenter; Williams.
Practice.—Wood Watson.
Materia Medica.—Wood's Theraputics; U. S. Dispensatory.
Surgery.—Miller; Chelius; Erichson; Druitt.
Obstetrics.—Cazeau; Meigs; Ramsbotham; Churchill.
Diseases of Women. — Colombot; Churchill; Bennett.
Diseases of Children. — Billard; Condie; Meigs.
Practical Anatomy.—Agnew; Dublin Dissector; Wilson's Dissector.
Beck or Taylor's Medical Jurisprudence.

### IOWA MEDICAL JOURNAL.

J. C. HUGHES } EDITORS. { WELLS R. MARSH.

This is the only Medical Journal published in the State. It is a work of 80 pages, and issued bi-monthly, for the sum of $2 00—invariably in advance. We are always thankful for subscribers and contributions.

